# Factors influencing the underutilization of mental health services among Asian American women with a history of depression and suicide

**DOI:** 10.1186/s12913-015-1191-7

**Published:** 2015-12-08

**Authors:** Astraea Augsberger, Albert Yeung, Meaghan Dougher, Hyeouk Chris Hahm

**Affiliations:** 1 Boston University School of Social Work, Boston, MA 02215 USA; 2 Depression Clinical and Research Program, Massachusetts General Hospital, Boston, MA USA; 3 Department of Psychiatry, Harvard Medical School, Boston, MA USA

**Keywords:** Asian Americans, Depression, Suicide, Help-seeking behaviors, Mental health utilization, Stigma, Women’s health

## Abstract

**Background:**

Despite the substantially high prevalence of depression, suicidal ideation and suicide attempts among Asian American women who are children of immigrants, little is known about the prevalence of mental health utilization and the perceived barriers to accessing care.

**Methods:**

The data were from the Asian American Women’s Sexual Health Initiative Project (AWSHIP), a 5-year mixed methods study at Boston University. The quantitative analysis examined the differential proportion of mental health utilization among 701 survey participants based on their mental health risk profile determined by current moderate to severe depression symptoms and lifetime history of suicidality. Mental health risk groups were created based on participants’ current depression symptoms and history of suicide behaviors: Group 1–low-risk; Group 2–medium-risk; Group 3–high-risk. Mental health care utilization outcomes were measured by any mental health care, minimally adequate mental health care, and intensive mental health care. The qualitative analysis explored the perceived barriers to mental health care among 17 participants from the medium and high-risk groups.

**Results:**

Among 701 participants, 43 % of women (*n* = 299) reported that they either suffered from current moderate to severe depression symptoms or a lifetime history of suicidal ideation or suicide attempt. Although the high-risk group demonstrated statistically significant higher mental health utilization compared to the low and medium-risk groups, more than 60 % of the high-risk group did *not* access any mental health care, and more than 80 % did *not* receive minimally adequate care. The qualitative analysis identified three underutilization factors: Asian family contributions to mental health stigma, Asian community contributions to mental health stigma, and a mismatch between cultural needs and available services.

**Conclusions:**

Despite the high prevalence of depression and suicidal behaviors among young Asian American women in the sample, the proportion of mental health care utilization was extremely low. The qualitative analysis underscores the influence of Asian family and community stigma on mental health utilization and the lack of culturally appropriate mental health interventions. Prevention and intervention efforts should focus on raising mental health awareness in the Asian American community and offering culturally sensitive services.

## Background

In 2010, there were approximately 18.2 million Asians in the U.S. population (U.S. Census Bureau), comprising 4.8 of the total American population. It is projected that by 2050 there will be 40.6 million Asians in the U.S. population, or 9.2 % of the total U.S. population [1]. Among subgroups of Asian Americans, young Asian American women have emerged as the highest risk in terms of mental health status. Specifically, Asian American women have the second highest suicide rate among the racial/ethnic groups in the U.S. for females 18–24 years old [2] and the incidence of suicide among Asian American women grew by almost 100 % between 2000 and 2009 [2, 3]. Despite the substantial risk of depression and suicide among young Asian American women, the current literature is limited in providing information about mental health utilization patterns in this population.

Evidence from the past decade demonstrates that Asian Americans suffering from mental illness delay or forgo mental health care. Alegria and colleagues reported that among Asian Americans diagnosed with any depressive disorder, two out of three (69 %) did not seek mental health treatment, compared to Hispanics (64 %), African Americans (59 %), and White Americans (40 %) [4]. Le Meyer and colleagues reported that among Asian Americans with a diagnosable mental health disorder, only 28 % used specialty mental health services (i.e., services delivered by a psychiatrist, psychologist, or other mental health professional) [5].

Multiple factors have been associated with the underutilization of specialty mental health services among Asian Americans including a delay in recognizing symptoms and seeking help [6], a lack of appropriate mental health providers, and the use of primary or alternative services such as religious leaders, family members, or peers for counseling [6–8]. Public stigma [9–13], language barriers (e.g., English speaking versus non-English speaking), and generational status were other factors associated with this underutilization [14–16].

Chu, Hsieh, and Tokars compared perceived need for help, help seeking behaviors, and types of help sought among Asian Americans and Latinos living in the U.S. who had reported a history of lifetime mental disorder, suicidal ideation, and suicidal attempts. Although Asian American and Latino samples were equally as likely to seek help from medical professionals, Asian Americans with mental disorders and suicidal ideation were significantly less likely than Latinos to perceive a need for help or to seek help. Asian Americans who sought help for suicidal ideation or attempts were more likely than Latinos to use non-professionals, including on-line support groups, self-help groups and hotlines. Chu, Hsieh and Tokers discuss the need for more research focused on the role of cultural factors in help seeking behaviors [14].

Using a nationally representative sample of Asian Americans, Abe-Kim and colleagues found that compared to third generation Asian Americans, Asian immigrants and second generation Asian Americans who are children of immigrants showed similarly lower patterns of mental health utilization and lower ratings on perceived helpfulness of treatment. These authors suggest that stigma may act as an important barrier to seeking mental health services among immigrants and children of immigrants [17], a finding supported by other studies [18, 19]. Eisenberg and colleagues reported that Asian and Asian American college students had the highest reported level of personal stigma among all the minority groups studied [20]. Ting and Hwang reported that Asian American college students who had lower levels of stigma tolerance (the ability to tolerate the cultural stigma associated with mental illness) were less likely to engage in help-seeking behaviors [2]. Chu and Sue noted that Asian Americans typically avoid mental health services because opting to utilize such resources requires admitting the existence of a mental health problem and may cause shame to the family if personal issues become public [21].

Three important limitations to the current literature require further investigation: samples have not been sufficiently targeted, acculturated women have not been studied, and the perceptions and experiences of high-risk women have not been solicited. First, the majority of studies examining perceived barriers to mental health utilization included Asian American samples reporting general psychological distress, rather than focusing specifically on samples with moderate to severe depression symptoms, history of suicidal ideation, or suicide attempts [5, 6, 17, 22, 23]. Given the high rates of depression and suicidality among young Asian American women, and given that a lifetime history of depression, suicidal ideation, and suicide attempts are significant predictors of completed suicide [24, 25], data from these at-risk samples must be generated to better understand the perceived barriers to mental health utilization. Second, studies focused on cultural stigma drew largely from samples of Asian American college students [2, 20], but did not specifically address immigration status. Research on Asian Americans shows that mental health utilization patterns among the 2nd generation or members of generation 1.5 are similar to those of 1st generation immigrants, and mental health utilization begins to increase among 3rd generation immigrants [17]. To date, no study has shed light on the barriers to mental health utilization among Asian American women who are acculturated (1.5 or 2nd generations). Third, previous research does not provide an in-depth exploration of the perceptions of and experiences with mental health utilization of the Asian American women of concern to us here: those who are at high risk for depression symptoms and suicide.

In this study, we addressed the research limitations by examining barriers to specialty mental health care utilization among 1.5 or second generation young Asian-American immigrant women reporting a history of depression symptoms, suicidal ideation, and suicide attempts. We examined the prevalence of accessing 1) any mental health care, 2) minimally adequate mental health care, and 3) intensive mental health care. We tested the extent to which mental health utilization differs by women who have a low, medium, or high mental health risk profile. Finally, we provided an in-depth exploration of the perceptions and experiences of mental health care utilization among 17 women belonging to the medium (Group 2) and high-risk groups (Group 3).

## Methods

The Institutional Review Board (IRB) of Boston University approved all procedures in this study. Informed consent was provided by each participant as required by the Boston University IRB.

### Data collection

The data were from a 5-year mixed-methods study at Boston University, the Asian American Women’s Sexual Health Initiative Project (AWSHIP). The mixed methods included survey data and in-depth semi-structured interviews. The survey data were collected from January 2010 to March 2011. To be eligible for AWSHIP, women had to be (a) single, (b) between18 and 35 years old, (c) self-identified as Chinese, Vietnamese, Korean, or a combination of these ethnic groups, (4) immigrants from generation 1.5 (women who were born in a foreign country and grew up in the U.S.) or second-generation immigrants (women who were children of immigrants), and (5) located within the greater Boston area. Outreach workers strived to maintain ethnic diversity in the sample of women selected and a balance between 1.5-generation women and second-generation women. International students or women whose parents did not live in the U.S. were excluded since the AWSHIP project was interested in the effects of acculturation on health risk behaviors.

AWSHIP reached out to 8 universities and 20 community organizations including various libraries, health, legal, and art organizations as well as a radio station. These organizations helped recruit participants and some of these universities and organizations offered private interview rooms that allowed a computer-assisted survey interview (CASI). Of the 804 women who were screened during the data collection period of 2010 to 2011, about 2 % (*n* = 17) were ineligible and 10 % (*n* = 83) never followed through in participating in the study. The final sample consisted 701 participants; the largest ethnic group was Chinese (51.5 %, *n* = 361), followed by Korean (21.7 %, *n* = 153), Vietnamese (19.5 %, *n* = 137), and mixed race (7.2 %, *n* = 50). The mean age of the quantitative sample was 22.5 years old.

Those who met the eligibility criteria completed a CASI. In order to accommodate a potential language barrier with English, CASIs were offered in 5 different languages: English, traditional Chinese, simplified Chinese, Korean, and Vietnamese. The multilingual CASI version was the product of a total of 12 translators’ and back translators’ work (two translators and two back translators each for Chinese, Korean, and Vietnamese). In the consent form for the CASI, women were asked if they would be willing to participate in a follow-up face-to-face interview.

Thirty-eight participants agreed and were interviewed regarding their family’s immigration history, the impact of American culture on their family, intimate relationships, sexual behaviors, mental health status, and mental health care utilization. The qualitative interviews were conducted by three Asian American women with mental health expertise: the principal investigator (PI) who is a Korean American Ph.D. social work researcher and clinician, a Korean American anthropologist, and a Chinese American Masters of Social Work (MSW) intern. All interviews were audio recorded and transcribed verbatim.

Due to the sensitive nature of the interview questions, a semi-structured interview guide was used. The interview guide ensured that general topics were addressed in each interview, but afforded the interviewer flexibility to probe deeper and/or focus more specifically on certain aspects of an interviewee’s response. For example, when discussing depression, the interviewer asked the question: “Was there ever a time in your life when you felt really down for a period of more than 2 weeks?” Depending on the interviewee’s response the interviewer would ask follow up questions pertaining to the stage of life, how long the low mood lasted, whether mental health services were utilized, and whether those services were helpful in addressing their depression symptoms.

The initial qualitative analysis showed that many women experienced multiple forms of adversity such as suicidal behaviors, drug use, and/or sexual abuse. To ensure that the interviews included a sufficient number of women who could describe the impact of acculturation and depression symptoms, we followed recommended guidelines [26–28] and oversampled women who had a history of adversity. Out of 38 women who participated in the qualitative interviews, a subset of 17 participants were selected for the qualitative analysis for this study. The largest ethnic group was Chinese (47 %, *n* =8), followed by Koreans (24 %, *n* =4), mixed race (17 %, *n* = 3) and Vietnamese (12 %, *n* =2). The average age of the qualitative sample was 22.0 years old. Participants were selected based on self-reporting in the CASI of current moderate to severe depression symptoms, with some reporting a lifetime history of suicidal ideation and/or suicide attempt. Participants belonged to either the medium-risk (Group 2) or the high-risk group (Group 3) based on their survey data. Interview participants were given pseudonyms 1–17 for confidentiality purposes.

### Data analysis

#### Quantitative measures

Moderate to severe depression scores were characterized based on The Center for Epidemiologic Studies Depression Scale (CESD), which has been validated for major depression and sensitivity among Asian adults in Asia [29, 30] as well as Asian women in the U.S. [31]. The CESD derives from a self-screening test that measures symptoms for a severe depressive episode for the last 2 weeks. Moderate to severe depression was examined by utilizing 20 questionnaires [32, 33]; total scores ranged from 0 to 46 and were dichotomized as no or minimal depressive symptoms (0 to 15) or moderate/severe depressive symptoms (16 to 46). This dichotomization has high internal consistency (Cronbach’s alpha = 0.90), and this categorization has been used in other studies [34].

History of suicidal ideation and suicide attempts over the last 12 months were measured by asking participants if they had seriously considered suicide and if they had attempted suicide, respectively. Participant responses were recorded as *yes* or *no* for both variables [35].

*The mental health risk groups* were created based on the participants’ current depression status and history related to their suicide behaviors: 1) the low mental health risk group was determined to be those who did *not* have current moderate to severe depression symptoms and *without a* lifetime history of suicidal ideation or suicide attempt (Group 1); 2) the medium mental health risk group was determined to be those who had *either* current moderate to severe depression symptoms or those who had a lifetime history of suicidal ideation or suicide attempt (Group 2); 3) the high mental health risk group was determined to be those who had *both* current moderate to severe depression symptoms and those who had a lifetime history of suicidal ideation or suicide attempt (Group 3).

*Mental health utilization* was indicated by: 1) Any mental health care, 2) minimally adequate mental health care, and 3) intensive mental health care.

*Any mental health care* consisted of attending at least one outpatient mental health counseling appointment in the last 12 months. This was ascertained by the question: “How many times have you been treated for any psychological or emotional problems in outpatient counseling in the past year?” Responses were dichotomized as 1 for attending at least one outpatient mental health counseling appointment and 0 for not attending any mental health counseling appointments.

Based on evidence-based treatment guidelines and recommendations from the American Psychiatric Association [36], eight or more visits to mental health counseling is considered *minimally adequate care* [37, 38]. However, because Asian-Americans have high dropout rates compared to other ethnic and racial groups [21], we also measured minimally adequate mental health care for those who attended at least four outpatient mental health visits in the past year.

*Minimally adequate mental health care* was measured by a positive response to either (1) attending at least 4 or (2) attending at least 8 outpatient mental health visits in the last 12 months. Responses were dichotomized as 1 for attending at least 4 outpatient counseling sessions in 1 year, and 0 for less than 4. Responses were also dichotomized as 1 for attending at least 8 outpatient mental health visits and 0 for less than 8.

*Intensive mental health ca*re consisted of receiving inpatient mental health or partial hospital care, or residential or day treatment in the last 12 months. This was ascertained by two questions; “How many times have you been treated for any psychological or emotional problems in an inpatient hospital (or partial hospitalization program) in the past year?” and “How many times have you been treated for any psychological or emotional problems in a residential or day treatment program in the past year?” Responses were dichotomized as 1 for “yes” to at least one question, and 0 otherwise.

### Statistical analysis

The quantitative analysis focused on the prevalence of mental health utilization among participants receiving: 1) any mental health care, 2) minimally adequate mental health care, and 3) intensive mental health care. Chi-square tests were used to compare the proportion of 4 different mental health care utilization outcomes among 3 groups of women based on their risk profile (low-risk, medium-risk, high-risk). For the statistical testing, *p* value of 0.05 was used to determine the statistical significance.

#### Qualitative measures

Interview data were analyzed using thematic analysis, reported by Braun and Clarke [39] as a common method of qualitative analysis in psychology and other mental health fields. The authors reviewed all interview transcripts and developed initial codes. The codes were then applied to additional data, expanded upon, and collated into three levels: family, community, and service. As recommended when analyzing qualitative data [40], the first author wrote memos describing common patterns and themes within each level, and providing direct quotes from participant transcripts. To enhance the reliability of the findings, the other authors reviewed the memos and discussed any discrepancies. Additionally, the findings were triangulated with the empirical literature.

Mental health stigma was identified as the central theme on the family and community levels. On the family level, the code “parents dismissive of mental health concerns” was used to describe instances when parents denied the woman’s mental health concerns because of the cultural stigma surrounding mental health. The code “parents’ emphasis on saving face” was used to highlight the pressure participants experienced from their parents to keep their mental health issues within the family unit in order to maintain a positive family image. At the community level, the code “community views mental health as a taboo subject” described the lack of awareness and acknowledgment of mental health issues in the Asian community. The code “community disapproves of burdening others with problems” referred to instances when participants chose not to discuss mental health issues to others so as not to burden them.

On the service level there was a mismatch between the services provided to participants through the U.S. mental health service system and those they perceived would be helpful. The code “lack of culturally appropriate intervention models” described the absence of evidence-based intervention models for this population. The code “women left alone to find alternative coping” described participants’ feelings of isolation resulting in using alternative coping mechanisms for managing their depressive and suicidal thoughts.

## Results

### Quantitative results

Table 1 shows the utilization patterns for the four groups: total sample (*n* = 701), the low-risk group (*n* = 401 women without current moderate to severe depression symptoms and without lifetime suicidal ideation/attempt); the medium-risk group (*n* = 226 women with either current moderate to severe depression symptoms or lifetime suicidal ideation/attempt), and the high = risk group (*n* = 73, women with current moderate to severe depression symptoms with lifetime suicidal ideation/attempt).

**Table 1:** Prevalence of mental health utilization patterns among women in low-risk (Group 1), medium-risk (Group 2), high-risk (Group 3) groups

Mental health utilization characteristics	Total sample	Low risk (G1)	Medium risk (G2)	High risk (G3)	Testing
		Women *without* current depression *or* lifetime suicidal ideation/attempt	Women with *either* current depression *or* lifetime suicidal ideation/attempt	Women *with* current depression *and* lifetime suicidal ideation/attempt	
	(*n* =701) *n*, %	(*n* =401) *n*, %	(*n* = 226) *n*, %	(*n* = 73) *n*, %	
Any mental health care	86/700^a^	43/401	49/225^a^	27/72^a^	*χ*^2^ = 36.2
	12.3 %	10.7 %	21.8 %	37.5 %	*p* = 0.0001
Minimally adequate mental health care (at least 4 times)	50/700^a^	16/401	20/225^a^	14/72^a^	*χ*^2^ = 23.4
	7.15 %	4 %	8.9 %	19.4 %	*p* = 0.0001
Minimally adequate mental health care (at least 8 times)	28/700^a^	13/401	13/225^a^	11/72^a^	*χ*^2^ = 17.8
	4 %	3.2 %	5.8 %	15.3 %	*p* = 0.0001
Intensive mental health care	24/701	15/401	25/226	13/72^a^	*χ*^2^ = 23.6
	3.4 %	3.7 %	11.1 %	18.1 %	*p* = 0.0001

*G1* refers to Group 1, *G2* refers to Group 2, and *G3* refers to Group 3

^a^In G1, G2, G3, there was one missing value in some of the mental health utilization outcomes

Among the total sample, 12.3 % of the women reported that they used any mental health care in the past 12 months. As expected, women in the high-risk group (Group 3) were statistically significantly more likely to receive any mental health care, minimally adequate mental health care (both 4 times and 8 times), and intensive mental health care, compared to those who were in the medium-risk group (Group 2) and the low-risk group (Group 1) (*p* = 0.001 for all the models). However, despite the fact that women in Group 3 received significantly more mental health services compared to those in Group 2 and Group 1, the overall mental health utilization for women in Group 3 was still low. For instance, more than 60 % of women in Group 3 who had current moderate to severe depression symptoms as well as a lifetime history of suicidal ideation did *not* receive any mental health care in the past 12 months. More than 80 % of Group 3 women did *not* continue in treatment long enough to attend at least 4 or 8 mental health visits. Further, more than 80 % of Group 3 women did *not* receive intensive mental health care, measured by treatment at an inpatient hospital or partial hospitalization program. This low mental health utilization pattern was also pervasive for women in Group 2.

### Qualitative results

The low rates of mental health utilization reported in the quantitative analysis, in contrast to the serious mental health concerns reported by participants, led the authors to examine the research question: What are the perceived barriers to mental health utilization among participants? All 17 participants belonged to either the medium-risk Group 2 or the high-risk Group 3. Nine women belonged to Group 2 and eight women belonged to Group 3.

**Fig. 1:**
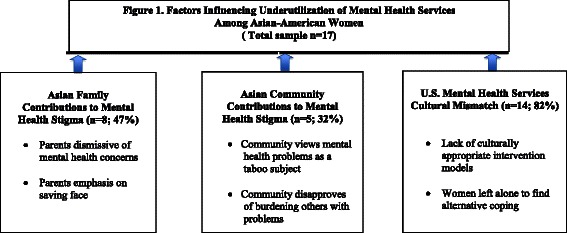
Factors influencing underutilization of mental health services among Asian-American women (total sample *n* = 17)

Figure 1 illustrates factors influencing the underutilization of mental health services among our participants: the family and community contributions to mental health stigma, and a mismatch between the U.S. mental health service system and the cultural needs of Asian American women. Without any specific questions relating to stigma, 47 % (*n* = 8) of interview participants reported family contributions to mental health stigma, 30 % (*n* = 5) reported community contributions to mental health stigma, and 82 % (*n* = 14) discussed the cultural mismatch between U.S. mental health services and the perceived needs of Asian American women.

### Asian family contributions to mental health stigma

#### Parents dismissive of mental health concerns

Five participants discussed parental attitudes as barriers to mental health utilization. They reported that parents dismissed their emotional concerns without taking the time to understand the nature of the concerns. A general theme among participants was that their families denied the existence of mental illness due stigma in the Asian culture. Participant 17 stated, “Umm, when I was in high school I was convinced that I was bi-polar. I’m actually almost positive that I am, but, umm, my parents don’t believe in mental illness.” Similarly, participant 12 reported that her parents dismissed her depression because they could not physically see anything wrong with her:

It’s like, “Okay, well, if you break your arm, you go see a doctor for help, right?” Well, if you’re not feeling like yourself emotionally or mentally or whatever it is, it’s like you need to seek help as well. It’s just that I feel like a lot of it is just that you can’t see it. It’s so—so much has to do with like not being able to visualize it or like seeing how much someone needs help. Yeah. I feel like that’s a lot with my—like with my parents. Like they don’t understand what I’m going through because they don’t see it.

Participants reported feeling lonely and isolated because they were unable to talk with their parents about their depression. Participant 4 reported feeling “emotionally abandoned” by her mother. Participant 9 reported, “every time I try to tell her how unhappy I am, ‘cause people keep telling me, they’re like, “Why don’t you tell your mom how unhappy you are and just let her know?” She just brushes it off like it’s nothing, you know, like it’s not important.”

#### Parents’ emphasis on “saving face”

Five participants reported the importance of maintaining familial privacy by not speaking to people outside of the family about mental health problems. Participant 2 discussed her mental health problems with her High School guidance counselor and her parents said, “Why would you say something like that, why did you tell people about our personal lives, we should keep it in the family.” In this instance the participant was willing to share her mental health issues with a professional, however her parents’ reaction led her to question her decision to talk with someone outside the family unit.

The above excerpt highlights the cultural value placed on “saving face.” Participants felt pressure from their parents to present to the outside world as if everything was okay. Admitting to having a mental health problem was perceived as being “weak,” as reflected in this statement by participant 3: “So they (her parents) kind of raised me with the kind of like, umm, I feel a certain kind of responsibility… So like I feel like I need to put on a strong front I guess.” In this instance the participant feels a responsibility to hide her mental health issues so she is perceived as strong and competent. Similarly, when participant 9 told her mother that she was hospitalized during college for a suicide attempt, her mother stated, “Oh, I don’t know how you can be so weak… You have to be strong.” Her mother went on to tell her daughter that at one point in her life she thought about committing suicide but would never go through with it because she “needed to be strong.”

### Asian community contributions to mental health stigma

#### Community views mental health problems as a taboo subject

Community level attitudes towards mental health problems served as barriers to the women’s mental health utilization. Participants reported that, in the Asian culture, there is a lack of awareness and/or acknowledgment of mental health issues. Participant 9 said, “I don’t know what it is. I don’t know if it’s like the culture that they don’t… you know, want to admit that…I’m mentally unstable or something.”

The lack of awareness and/or acknowledgement of mental health problems in the Asian culture make it a taboo subject. Participants reported feeling discouraged from seeking help from a therapist. Participant 12 stated, “I know there’s like a stigma about like, you know, like with Asian people like seeing—like, uh, having a therapist or talking to a psychologist.” In this instance, the participant sought professional help but did not discuss it with her family or friends. Similarly, Participant 11 noted that there is a stigma in Asia surrounding going to counseling, so she would seek services only if it was kept confidential.

#### Community disapproves of burdening others with problems

Participants reported not wanting to burden friends and family by openly discussing mental health issues. Participant 6 said, “Cause I feel like… I, I never wanted to burden other people with those problems. So even my closest friends—like they would know what was going on, but not to the extent that… not to a great extent at all.” Participant 4 noted that she wanted to talk with Asian people at her church about her depression but she didn’t want to bother them, nor did she feel as if they would understand. Participant 4 also noted that her non-Asian friends would not be able to understand the stigma surrounding mental health in the Asian community: “cause I felt like my American friends didn’t really understand the dilemmas that I was having. And how, like, I was struggling with, like, my culture, doing the stuff that I was doing. And, like, my female American friends would be just like, “Girl, just whatever”’.

### U.S. mental health service system–mismatch between cultural needs and available services

The majority (82 %) of interview participants identified service level factors influencing help seeking behaviors, including the failure of professionals to understand and acknowledge the stigma surrounding mental health concerns in the Asian community, the lack of a more holistic approach, and the limited number of dual-culture practitioners.

#### Lack of culturally appropriate intervention models

Ten participants reported that the current talking-therapy model lacks a culturally sensitive approach. They referred to “talk therapy” as not being helpful in addressing mental health issues. Participant 8 said, “I personally didn’t like the mode of therapy that was available at the time. It’s like a lot of talk therapy which… I’m kind of like more of a… I’m kind of a do-it-yourself-er.” Participant 1 reported, “Well I don’t really like going to therapists because like I’ve had two and–After like the first time when I pretty much just told them everything— Like it would be like sitting in a room with them for 30 min and then just asking me questions and it’s all I think about is like my parents are spending 20 dollars a minute for me to be here and 5 min— Has passed and I haven’t—nobody said a word and that’s all I think about during those sessions so and like, I don’t know. I didn’t like it.”

Participant 14 discussed the value of the holistic approach to treatment at the counseling center, which incorporated the mind and body, as opposed to the approach at the mental health center, which was focused on psychiatric diagnosis and treatment. Referring to the benefits of the counseling center, participant 14 stated, “I think the focus is more, umm, on like personal well-being and sort of like looking at—looking at the whole person.”

Participant 11 emphasized that it was important to have a practitioner who understood the “dual-culture” experience of being raised in the U.S. but also being influenced by Asian family and community attitudes towards mental health. Such an understanding would help practitioners provide services that were tailored to individual needs. When discussing the lack of mental health resources for Asian women, participant 4 reported, “I would have loved to connect with like more Asian women, to like talk about this stuff.” Similarly, participant 3 highlighted the need for more Asian mentors and counselors.

#### Women left on their own to find alternative coping

Participants reported using alternative coping methods to manage depression symptoms and suicidality. Examples included reading self-help books and searching the Internet for strategies to deal with mental health concerns. Participants perceived these sources to be beneficial because they were confidential and provided concrete information, teaching participants *how* to manage mental health symptoms. Participant 1 opted to use self-help books, stating, “Because it like tells you like how to be happy–And like how to not be depressed, whereas like I feel like with a therapist, all you do is talk.” Participant 8 reported searching on line for help with depression, stating, “I would just look up ways that people deal with depression.”

Nine participants reported using more risky coping methods, such as alcohol or drugs, to manage depressive symptoms. Participant 8 stated, “I would literally use alcohol to feel better about a bad experience.” Participant 11 stated, “I used alcohol when I was unhappy. I realized it’s a really bad idea because it just intensified the bad feeling I had before I drank.” Participant 9 reported smoking marijuana daily to cope with depression: “last year, like I was staying at home and depressed, I used it (marijuana) like almost every day.”

When discussing reasons for using substances, participants reported that substances numbed the pain and/or made them feel temporarily happy. Participant 17 said, “So, like if I’m sad and I do painkillers, I definitely feel, like, way, way, way better.” Similarly, participant 2 discussed her use of heroin, “It was just like you’re normal, but it just—s—or like, I’m going to cut off that part of your brain that makes you sad. So you’re just really, really happy. And I—it—I—it—it’s like a pain killer too.”

In sum, 82 % of interview participants discussed the lack of culturally appropriate mental health interventions available to them. Some participants sought information and support from alternative sources such as self-help books and the Internet. Other participants discussed risky coping methods, such as alcohol or substances, to self-medicate and/or numb their psychological pain.

## Discussion

To our knowledge, this is the first study to use quantitative data to show mental health care utilization patterns among Asian American female children of 1.5 or 2nd generation immigrants who have suffered from current moderate to severe depression symptoms or have experienced lifetime suicidal ideation or attempts. Our results demonstrate a severe underutilization of mental health services by Asian American women compared to other ethnic groups, as supported by Alegria and colleagues [4].

To our knowledge, no previous studies have examined the barriers to mental health care utilization among Asian children of immigrants who have experienced depression symptoms, suicidal ideation, and suicide attempts. Our quantitative results demonstrate that mental health utilization in the past 12 months by both medium-risk Group 2 and high-risk Group 3 were low. Only 22 % of the medium-risk group (Group 2 who had *either* current moderate to severe depression symptoms or a lifetime history of suicidal ideation or suicide attempt) had used any mental health care in the past 12 months. Although the high risk group (Group 3 who had *both* current moderate or severe depression symptoms and a lifetime history of suicidal ideation or suicide attempt) were statistically significantly more likely to use any mental health care, minimally adequate mental health care, and intensive mental health care compared to the low risk or medium risk groups, less than 20 % of them reported minimally adequate mental health services and intensive mental health care. These patterns of mental health utilization are also much lower than those of other ethnic/racial groups in the U.S. who experience similar mental health conditions [37]. This reflects a severe underutilization of mental health care.

Our qualitative results provide some insight into the alarmingly low patterns of mental health utilization among Asian women at risk for depression and suicidality. We found that family and community stigma attached to mental health problems and mental health services influenced our participants’ utilization of mental health services. Even though our participants were either born in the U.S. or immigrated when they were children, the cultural stigma towards receiving mental health treatment operated as a barrier for accessing the mental health care they needed. By delving into cultural stigma among depressed and suicidal daughters of immigrant Asian Americans, our study provides evidence that it is not sufficient to understand the health care utilization patterns of populations such as this one without incorporating stigma as an explanatory factor and one which lends itself to action if we wish to rectify underutilization. Furthermore, while the current literature on stigma discusses the fact that the presence of stigma impacts access to healthcare resources [20, 21], our study provides a more detailed analysis. It describes how stigma acts as a barrier on family and community levels, blocking help-seeking among women in need. Although there is literature noting that stigma has an impact on utilization of mental health services among Asian American women, the majority of these studies focused on immigrants [41, 42] or specific Asian ethnicities [10, 12, 16, 23]. Other studies examining Asian Americans as a whole did not disaggregate the immigration generation [2, 6, 7, 11, 43]. In contrast, we focus on Asian American female children of 1.5 or 2nd generation immigrants who have suffered from current moderate to severe depression symptoms or have experienced lifetime suicidal ideation or attempts.

Our study provides new insights regarding the access to and adequate use of mental health services among depressed or suicidal children of immigrants. This will provide a better foundation for researchers to develop solutions to increase mental health utilization for this at- risk group of women. This finding raises questions about health care utilization models that omit or minimize the role of cultural factors. For example, Andersen’s behavioral model has been a prevailing model in the healthcare utilization literature explaining individuals’ decisions to seek healthcare services based on specific predisposing (demographic status, social structure, and health beliefs), enabling (personal, familial, or community resources), and need (perceived and evaluated need of health services) factors [44, 45]. Andersen’s model has been criticized for not paying enough attention to the role of cultural factors and the cultural competence of health care systems. This study gives weight to such critiques.

While our study is exploratory, it has important clinical implications. First, it is essential for clinicians to be aware of how difficult it is for Asian-American women with a history of major depression, suicidal ideation, and suicide attempts to seek mental health services due to the role of stigma. Second, given the pervasiveness of stigma in family and community attitudes and behavior, innovative methods and venues are needed to screen for depression symptoms and identify suicidal young Asian-American women. One example would be to perform depression screenings at primary care clinics, actively approaching patients who screen positive for depression symptoms, and providing on-site culturally sensitive treatment [46]. Since Asian Americans are open to obtaining care from medical professionals [14], and receiving mental health treatment through a primary care physician, this is likely to reduce the cultural stigma of seeking help. This integration of mental health services within the primary care setting could be an alternative treatment option.

Participants discussed their desire for more holistic services focused on mind-body integration. While they were reticent to participate in mental health services, they were open to services focused on health and wellness. Thus, sending clients to wellness centers that provide mind body treatments (i.e., yoga, tai chi, qigong, mindfulness training) may reduce the stigma of seeking help [47]. Studies demonstrate that these modalities have been effective in treating mild and moderate depression symptoms and may serve as adjunctive treatment for more conventional care [48–50].

Participants discussed the use of self-help books and the Internet for information focused on depression and suicidality. Thus, another possible intervention would be providing clients with tele-therapy [51], which would allow them access to mental health services in the privacy of their homes. It would be important that the professionals providing this service understand the dual-culture identity of women raised in the U.S. but influenced by Asian attitudes and values.

Finally, given our finding of the family and community contributions to mental health stigma, a mental health awareness campaign should target Asian family members, peers, and community agencies. Culturally sensitive screening and treatment services that incorporate cultural aspects, reduce stigma and educate about mental illness may eventually reduce the record high rates of suicide in the U.S. for this vulnerable young Asian-American female population.

### Limitations

Our study has some limitations. First, due to stigma surrounding mental health among Asian Americans, participants may have underreported the prevalence of mental health problems, or their help-seeking behaviors. Second, we did not include a stigma variable in the CASI, so we were limited in measuring the impact of stigma on mental health utilization. Third, we did not include questions pertaining to stigma in our qualitative interviews; therefore, we were limited in terms of the depth of information collected. Nevertheless, it is impressive that, without specific questions to elicit responses about stigma, virtually all our interviewees spontaneously volunteered that stigma played a role in their behavior related to mental health problems and help-seeking. These responses suggest that future studies should include a stigma variable in their analysis as well as more specific questions focused on family and community stigma as a potential barrier to mental health utilization.

## Conclusions

Although the sample of Asian American women had a high prevalence of depression symptoms and suicidal behavior, mental health care utilization was remarkably low. The qualitative analysis underscores the influence of Asian family and community stigma on mental health utilization. The qualitative results also highlight the perceived lack of culturally appropriate mental health interventions for this population. Prevention and intervention efforts should focus on reducing the stigma associated with mental health problems in the minds of Asian family and community members and creating culturally sensitive mental health interventions.
